# Triglyceride-glucose index as a marker in cardiovascular diseases: landscape and limitations

**DOI:** 10.1186/s12933-022-01511-x

**Published:** 2022-05-06

**Authors:** Li-Chan Tao, Jia-ni Xu, Ting-ting Wang, Fei Hua, Jian-Jun Li

**Affiliations:** 1 The Third Affiliated Hospital of Soochow University, Juqian Road, Changzhou, 213000 China; 2 State Key Laboratory of Cardiovascular Diseases, Fu Wai Hospital, National Center for Cardiovascular Diseases, Chinese Academy of Medical Sciences and Peking Union Medical College, No 167 BeiLiShi Road, XiCheng District, Beijing, 100037 China

**Keywords:** Triglyceride-glucose index, Insulin resistance, Cardiovascular disease

## Abstract

The triglyceride-glucose (TyG) index has been identified as a reliable alternative biomarker of insulin resistance (IR). Recently, a considerable number of studies have provided robust statistical evidence suggesting that the TyG index is associated with the development and prognosis of cardiovascular disease (CVD). Nevertheless, the application of the TyG index as a marker of CVD has not systemically been evaluated, and even less information exists regarding the underlying mechanisms associated with CVD. To this end, in this review, we summarize the history of the use of the TyG index as a surrogate marker for IR. We aimed to highlight the application value of the TyG index for a variety of CVD types and to explore the potential limitations of using this index as a predictor for cardiovascular events to improve its application value for CVD and provide more extensive and precise supporting evidence.

## Introduction

Cardiovascular disease (CVD) is a leading cause of morbidity and mortality worldwide, posing serious public health challenges and placing an economic burden on patients [1]. Although several risk factors for CVD have been established, including age, male sex, obesity, hypertension, hypercholesteraemia, and diabetes, recent studies have demonstrated that some individuals without these risk factors may also develop CVD [2, 3]. Additionally, despite the development of advanced techniques and the popularization of primary and secondary prevention measures, patients with CVD remain at increased risk of recurrent adverse cardiovascular events [4]. Therefore, identifying persons at early risk for CVD will have remarkable clinical significance for improving risk stratification and therapeutic management.

Insulin resistance (IR) is a state of decreased sensitivity and responsiveness to the action of insulin and has bee identified as a hallmark of T2DM, even preceding diabetes for several years [5]. There has been increasing evidence demonstrating that IR and related disorders contribute to the development of CVD in diabetic as well as nondiabetic subjects [6]. It is well known that individuals with IR are predisposed to developing several metabolic disorders, such as hyperglycaemia, dyslipidaemia, and hypertension, all of which are strongly associated with poor outcomes of CVD [7]. Thus, IR has been regarded not only as a pathogenic cause but also as a predictor of CVD in both general populations and subjects with diabetes. Therefore, developing convenient and reliable screening tools to detect IR and predict cardiovascular risks is of particular importance.

Currently, there are no specific methods for the accurate determination of IR. The gold standards of the euglycemic insulin clamp and intravenous glucose tolerance testing are invasive and expensive; although they are used in academic studies, they are not applied in clinical practice [8]. The homeostasis model assessment-estimated insulin resistance (HOMA-IR) index, a means for detecting β-cell function and IR, is widely used at present, but it has limited value in subjects receiving insulin treatment or those who do not have functioning beta cells [8]. To address this limitation, the triglyceride-glucose (TyG) index has been developed and was shown to be superior to HOMA-IR in assessing IR in individuals with and without diabetes [9]. According to previous studies, this simple, convenient, and low-cost surrogate does not require insulin quantification and may be used in all subjects regardless of their insulin treatment status [10]. Furthermore, recent studies have demonstrated that the TyG index is an independent predictor of prognosis in diabetic or nondiabetic patients with CVD, suggesting its potential clinical utility in predicting cardiovascular risk.

In this review, we systemically describe the history of the TyG index as a marker for IR. We will also discuss recently published literature that has helped to shed light on the application value of the TyG index in a variety of CVD settings, as well as its potential underlying mechanisms associated with CVD. Additionally, the limitations of the TyG index in predicting CVD are also discussed.

## Methods

This systematic review examined the application value of the TyG index in a variety of CVD types. Study selection included cross-sectional, case–control, or retrospective studies involving clinical populations with different CVD phenotypes. There were no language or time restrictions for eligible studies. The electronic databases PubMed and Web of Science were used. The search terms used were “TyG index” OR “triglyceride-glucose index” AND “coronary artery disease” OR “acute coronary syndrome” OR “in-stent restenosis” OR “arterial stiffness” OR “coronary artery calcification” OR “heart failure”. Screening of the retrieved titles and/or abstracts was performed in duplicate using Endnote Software, Version X8, and eligible studies were identified. Two authors (Li-chan Tao and Jia-ni Xu) retrieved the full texts of these studies and assessed them for eligibility. Disagreements were resolved through discussion.

## History of the use of the triglyceride-glucose index

The TyG index, calculated as TyG index = ln [Fasting triglyceride (mg/dl) × fasting glucose (mg/dl)]/2, is a composite indicator composed of fasting triglyceride (TG) and fasting glucose (FG) levels. It was first proposed in 2008. In a large cross-sectional study of apparently healthy individuals, the TyG index was found to be a better surrogate (sensitivity 84.0% and specificity 45.0%) to identify IR than the HOMA-IR index [9]. However, its low specificity (45.0%) and potentially high proportion of false-positive tests has limited the widespread use of the TyG index in screening for IR. In 2010, a cross-sectional study involving 99 individuals with various degrees of body weight and glucose tolerance was performed by Guerrero-Romero et al., and they identified the TyG index as an optimal tool for the assessment of IR, showing high sensitivity (96.5%) and specificity (85.0%) compared to the gold standard, the euglycemic-hyperinsulinaemia clamp test [11]. Furthermore, in a cross-sectional study of 82 Brazilian subjects with T2DM or normal glucose tolerance conducted in 2011, the TyG index was confirmed to be a better marker for estimating IR than the HOMA-IR index (area under the ROC curve (AUC): TyG index: 0.79, HOMA-IR index: 0.77) [12]. However, since both studies had small sample sizes, the results were not fully convincing.

Since then, the TyG index has been proven to be a reliable and accessible index for evaluating IR in high-risk individuals by large clinical studies. IR plays a crucial role in the development of impaired glucose tolerance and diabetes mellitus (DM). In 2014, a study by Lee et al. enrolled a total of 5,354 middle-aged nondiabetic Koreans for long-term follow-up to assess diabetes status. They found that the risk of diabetes onset in the highest quartile of the TyG index was more than fourfold higher than that in the lowest quartile (relative risk, 4.095; 95% CI 2.701–6.207), suggesting that the TyG index might be a useful marker for identifying subjects at high risk of developing diabetes. In addition, this study revealed that the predictive power of the TyG index was better than that of the HOMA-IR index for evaluating IR [13]. However, the lack of positive comparisons for diagnosing DM limited their conclusions regarding the reliability of the TyG index in predicting the occurrence of DM. Then, in 2016, a study by David et al. revealed that the TyG index had better predictive power (AUC: 0.75, 95% CI 0.7–0.81) in diagnosing subjects with DM than fasting blood glucose (FBG) measurement (AUC: 0.66, 95% CI 0.60–0.72) and TG levels (AUC: 0.71, 95% CI 0.65–0.77) among 4820 individuals [14]. Thus, the TyG index may help to identify individuals at risk of developing DM in the future so that early interventions can be provided.

**Fig. 1:**
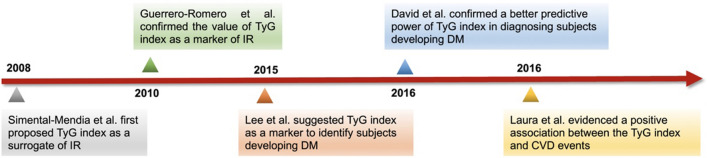
The useful history of triglyceride-glucose index (TyG). TyG: triglyceride-glucose index; IR: insulin resistance; DM: diabetic mellitus; FBG: fasting blood glucose; CVD: cardiovascular disease

In addition to DM, IR is also a significant hallmark of obesity, hypertension, dyslipidaemia (hypertriglyceridaemia and decreased high-density lipoprotein (HDL)), as well as other metabolic syndrome (MetS) symptoms [15, 16]. These metabolism-related components have been proven to be independent risk factors for CVD [17–19]. As a useful surrogate of IR, the TyG index has been gradually linked to the development of CVD and poor outcomes. Using a large sample from the Vascular Metabolic CUN cohort (VMCUN cohort) with a median period of 10 years of follow-up, Laura et al. first suggested a positive association between the TyG index (AUC: 0.708, 95% CI 0.68–0.73) and CVD events, including coronary heart failure (CHD), cerebrovascular disease, and peripheral arterial disease, independent of confounding factors[20]. Since then, the relationship between the TyG index and different types of CVD has been consecutively revealed (Fig. 1).

## TyG index in cardiovascular diseases (Fig. 2, Table 1)

**Fig. 2:**
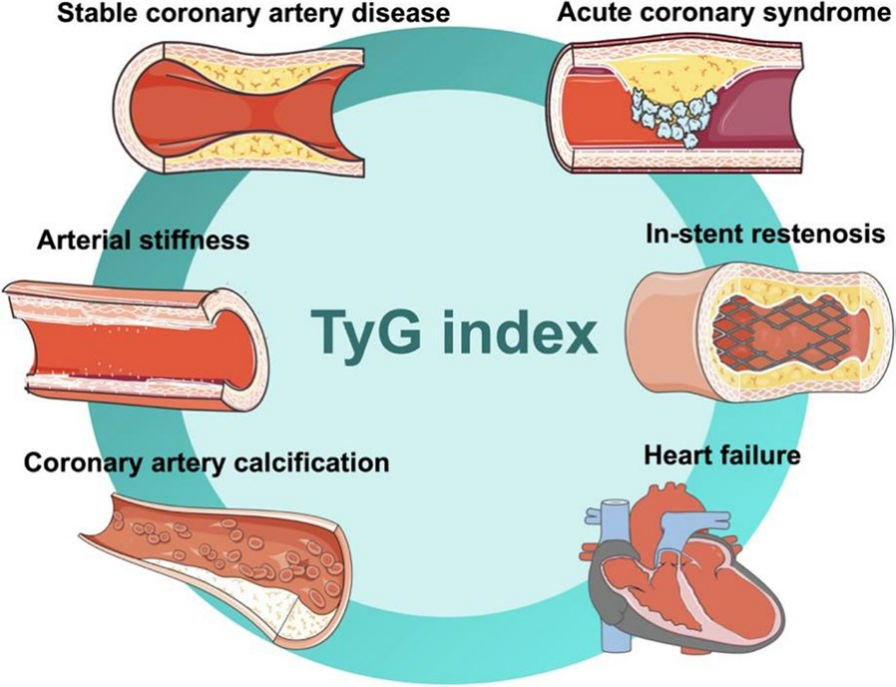
The application of triglyceride-glucose (TyG) index in cardiovascular diseases. TyG: triglyceride-glucose index

**Table 1:** Characteristics and results of studies regarding TyG index in cardiovascular diseases

Author, year, and country	Study design and population	Outcomes evaluated and evaluation methods	Adjusted variables	Key findings
Stable Coronary artery disease				
Jin, 2018, China [22]	Case–control, 1282 T2DM with new-onset, stable CAD	Incidence of MACCEs during follow up	Age, sex, body mass index, hypertension, family history of CAD, smoke, HDL-C, non-HDL-C, creatinine, UA, hsCRP, Gensini score	TyG index was associated with increased risk of MACCEs (HR: 1.693, 95% CI: 1.238–2.316)
Jin, 2018, China [23]	Case–control, 3745 patients with stable CAD	Incidence of CVEs	BMI, LVEF, hypertension, DM, UA, smoke, hsCRP, HDL-C, LDL-C	TyG index was positively associated with CVES (HR: 1.364, 95% CI: 1.100–1.691)
Gao, 2021, China [24]	Observational study, 1093 CAD patients with CTO lesion	Coronary collateralization grading evaluated by Rentrop score	Age, sex, BMI, hypertension, hypercholesterolemia, T2DM, smoke, stroke, UA, monocyte count, hsCRP	TyG index was correlated with the occurrence of impaired collateralization (OR: 1.59–5.72) in the T2 and T3 group compared with the first tertile group
Lee, 2016, Korea [25]	observational Study, 888 asymptomatic adults with T2DM	CAS evaluated by coronary CT angiography	Age, sex, glycemic control, blood pressure, LDL-C, medication	TyG index was an independent risk factor for CAS (OR:3.19, 95% CI: 1.371–7.424)
Si, 2021, China [79]	Observational study, 697 asymptomatic patients	CACS evaluated by coronary CT angiography	Age, smoke, hypertension, DM, LDL-C	TyG index was an independent risk factor for CAD (OR:2.007, 95% CI: 1.066–3.780)
Thai, 2020, Vietnam [26]	Cross-sectional observational study, 166 patients with T2DM	CAS evaluated by coronary CT angiography	Duration of diabetes, BMI, eGFR, physical activity, smoke, HbA1c, blood pressure	TyG index threshold at 10 resulted in 57% sensitivity and 75% specificity for predicting the presence of CS ≥ 70%
Park, 2020, Korea [29]	Retrospective, observational study, 1250 asymptomatic individuals without traditional CVRFs	CAS evaluated by coronary CT angiography	Age, sex, blood pressure, BMI, LDL-C, HDL-C, UA	TyG index had an incremental impact on CAD (OR: 1.473, 95%CI: 1.026–2.166)
Silva, 2019, Brazil [80]	Observational study, 2330 at least had one CVD in the last 10 years	Evaluation of risk factors for CAD	Sex, age, medication, presence of disease history	TyG index was positively associated with a higher prevalence of symptomatic CAD
Yang, 2021, China [81]	Prospective observational study, 5489 nondiabetic patients after PCI	Incidence of MACCEs during follow up	Age, sex, previous PCI/CABG, LVEF, HbA1c, TG, hsCRP	TyG index was not independently related to MACE for nondiabetic patients who underwent PCI (HR: 0.77, 95%CI 0.56–1.16)
Si, 2021, china [82]	Observational study, 680 CAD with T2DM, 985 non-CAD without T2DM	Identification of risk factors for CAD with T2DM	BMI, smoke, blood pressure, DM, ischemic stroke	TyG index was an independent risk factor for CAD with T2DM (OR:2.641, 95% CI: 1.154–6.045)
Chen, 2022, China [83]	Observational study, 1578 diabetic patients with OPCABG	Incidence of MACCEs during follow up	CKD, preoperative LVEF, insulin dependence, LDL-C, HDL-C, extent of CAD, left main disease, use of arterial graft, complete revascularization, use of IABP	TyG index was significantly and positively associated with MACCEs after OPCABG in patients with T2DM (OR:2.133, 95% CI: 1.347–3.377)
Acute coronary syndrome				
Wang, 2020, China [32]	Retrospective, observational study, 2531 ACS patients with diabetes	New-onset MACEs during 3-year follow up	Age, sex, smoke, previous MI and CABG, BMI, LVEF, multi-vessel disease, left main disease, HbA1c, hsCRP, statin and insulin use	TyG index was an independent predictor of MACEs (HR:1.455, 95% CI: 1.208–1.753)
Luo, 2019, China [33]	Retrospective, observational study, 1092 STEMI patients	Incidence of MACCEs during follow up	Age, blood pressure, smoke, platelet counts, albumin, HbA1c, UA, eGFR, three-vessel disease, number of stents	TyG index was positively associated with an increased risk of MACCEs (HR:1.529, 95% CI: 1.001–2.061)
Mao, 2019, China [34]	Observational study, 438 patients with NSTE-ACS	Incidence of MACEs during follow up	Age, Mets, LDL-C, HDL-C, eGFR, Killip class, GRACE score, CRP	TyG index was an independent predictor of the occurrence of the MACEs (HR:1.878, 95% CI: 1.130–3.121)
Zhang, 2022, China [35]	Observational study, 1655 nondiabetic patients with ACS with LDL-C < 1.8 mmol/l	Incidence of MACEs during follow up	Multi-vessel disease, PCI/CABG	TyG index was positively associated with revascularization (HR: 1.67, 95% CI: 1.02–2.75)
Drwita, 2021, Poland [84]	Observational study, 1340 nondiabetic patients with AMI	Incidence of MACEs during1-yaer follow up	CAD, eGFR, LDL-C, TC	TyG index was not independently related to MACE for nondiabetic patients who underwent PCI
Gao, 2021, China [36]	Prospective, observational study 1179 MINOCA patients	Incidence of MACEs during follow up	Age, sex, MI type, hypertension, diabetes, dyslipidemia	TyG index was significantly associated with an increased risk of MACE (HR: 1.33, 95% CI: 1.04–1.69)
Guo, 2022, China [85]	Retrospective, observational study 2030 prediabetic patients with ACS	Incidence of MACCEs during follow up	Age, sex, BMI, blood pressure, smoke, LDL-C, HDL-C, Cr, UA, eGFR, BNP, CRP, DM, hypertension	TyG index was significantly associated with an increased risk of MACCE (HR: 3.256, 95% CI: 2.618–4.749)
Jiao, 2022, China [86]	Observational study 662 ACS patients over 80 years old	Incidence of all cause of death and MACEs during follow up	Age, gender, BMI, blood pressure, LVEF, Gensini score, hypertension, diabetes, DM, previous MI, previous stroke, CKD, current smoking, TC, LDL-C, HDL-C, eGFR, UA, medication, LM lesion, multivessel lesion and treatment	TyG index is an independent predictor of long-term all-cause mortality (HR: 1.64, 95% CI: 1.06–2.54) and MACE (HR: 1.36, 95% CI: 1.05–1.95)
Zhao, 2021, China [87]	Observational study 1510 NSTE-ACS patients received elective PCI without diabetes	Incidence of MACEs during follow up	Age, gender, BMI, smokie, hypertension, dyslipidemia, previous history of MI, PCI, stroke, PAD, LM disease, three-vessel disease, chronic total occlusion, diffuse lesion, in-stent restenosis, SYNTAX score, treatment of LM, LCX, RCA, DES implantation, DCB application, complete revascularization, number of stents	TyG index is an independent predictor of MACE (HR: 2.433, 95% CI 1.853–3.196)
Ma, 2020, China [88]	Observational study 776 ACS with T2DM patients received PCI	Incidence of MACEs during follow up	Age, BMI, cblood pressure, HDL-Cl, HbA1c, sex, smoke, drinking, presence of peripheral artery disease, chronic kidney disease, cardiac failure, previous myocardial infarction, past percutaneous coronary intervention, medication, coronary artery disease severity, presence of lesions > 20 mm long, use of drug-coated balloon, complete revascularization	TyG index is an independent predictor of MACE (HR:2.17, 95% CI: 1.45–3.24)
Yang, 2022, China [89]	Retrospective, observational study 549 STEMI with NOAF patients	Incidence of MACEs during hospitalization and follow up	Age, hypertension, DM, BMI, Hb, WBC, neutrophil, platelet, glucose, TG, TC, HDL-c, LDL-c, eGFR, Left atrium diameter, LVEF, SS, Stent length, Killip class ≥ II	TyG index is an independent predictor of NOAF during hospitalization (OR: 8.884, 95% CI: 1.570–50.265)
Zhao, 2021, China [90]	Observational study 274 STEMI patients over 18 years old received OCT	Incidence of MACEs during follow up	Age, sex, LVEF, smoke, hypertension, hyperlipidemia, DM; Cr, heart rate, CRP	The middle tertile of TyG was associated with greater rates of MACEs in patients with PR but **not** in those with PE (HR: 6.01; 95% CI: 1.25–28.88)
Zhang, 2021, China [91]	Observational study 1932 ACS patients with T2DM	Incidence of MACCEs during follow up	Age, sex, BMI, blood pressure, medical history, smoke, duration of diabetes	TyG index is an independent predictor of MACCES in patients with T2DM and ACS (OR: 2.32, 95% CI: 1.92–2.80)
Zhang, 2020, China [92]	Observational study 3181 ACS patients	Incidence of MACEs during follow up	Age, gender, DM, hypertension, previous AMI, hemoglobin, albumin, eGFR, TGs, LVEF, multi-vessel/ left main coronary artery	TyG index was positively associated with MACEs (HR:1.19, 95% CI: 1.01–1.41) in patients with AMI
Hu, 2020, China [93]	Observational study 9285 ACS patients received PCI	Incidence of MACEs during follow up	Age, sex, smoke, hypertension, previous MI, previous stroke, previous PCI, previous CABG, ACS status, medication	High TyG index had a significantly greater risk of cardiovascular events regardless of diabetes status (HR:1.92, 95% CI: 1.33–2.77)
Zhao, 2020, China [94]	Observational study 798 NSTE-ACS patients with diabetes received PCI	Incidence of MACEs during follow up	Age, sex, BMI, blood pressure, smoke, drinking, duration of diabetes, dyslipidemia, prior MI, PCI, stroke, PVD, TC, HDL-C, eGFR, HbA1c, LVEF, SYNTAX score, LM treatment, DCB use, complete revascularization and number of stents, medication	TyG index was independently associated with higher risk of MACEs in NSTE-ACS patients with diabetes
In-stent restenosis				
Zhu, 2021, China [39]	Retrospective study, 1574 ACS patients with DES-based PCI	Incidence of ISR evaluated by follow-up angiography	Age, sex, BMI, LVEF, hsCRP, hypertension, DM, previous PCI, SYNTAX score, target vessel in LAD or RCA, application of intracoronary imagine; DES-sirolimus; total length of stents, and minimal stent diameter	TyG index was positively associated with DES-ISR (OR: 1.424, 95% CI: 1.116–1.818)
Arterial stiffness				
Lambrinoudaki, 2018, Greece [44]	Cross-sectional study, 473 non-diabetic postmenopausal women, without overt CVD	Carotid IMT, flow-mediated dilation of the brachial artery, PWV evaluated by ultrasound image	Mets, age, BMI, LDL-C, smoke, hypertension	TyG index was associated with carotid atherosclerosis and AS in lean postmenopausal women (OR: 3.119, 95% CI: 1.187–8.194)
Lee, 2018, Korea [45]	Observational study, 3587 healthy subjects	AS evaluated by baPWV	Age, blood pressure, BMI, LDL-C, HDL-C, DM, menopause	TyG index was significantly associated with increased baPWV (OR: 2.92, 95% CI: 1.92–4.44 in men, OR: 1.84, 95% CI: 1.15–2.96 in women)
Won, 2018, Korea [46]	Cross-sectional study, 2560 subjects without CAD, stroke, and malignancies	AS evaluated by baPWV	Age, sex, blood pressure, abdominal obesity, HDL, smoke, DM	TyG index was independently related to the baPWV (β = 0.158)
Su, 2021, China [47]	Cross-sectional study, 2035 subjects over 60 years old	AS evaluated by baPWV	Age, sex, BMI, waist circumference, SBP, DBP, TC, HDL-C, LDL-C, UA, eGFR, smoke, drinking status, CAD, hypertension, DM, anti-platelet agents, anti-hypertensive agents, hypoglycemic therapy, lipid-lowering therapy	TyG index was positively associated with baPWV (OR: 1.32, 95% CI: 1.09–1.60)
Li, 2020, China [50]	Observational study, 4718 patients with hypertension	AS evaluated by baPWV	Age, sex, education, BMI, waist circumference, physical activity, smoke, current drinking, blood pressure, UA, serum homocysteine, HDL-C, LDL-C, eGFR, DM, antihypertensive drugs, antiplatelet drugs	TyG index was positively associated with baPWV (OR: 2.12, 95% CI: 1.80–2.50)
Nakagomi, 2020, Japan [51]	Observational study, 1720 healthy men and 1098 healthy women aged between 25 and 55 years	AS evaluated by baPWV	Age, BMI, blood pressure, HbA1c, FBG, LDL-C, HDL-C, UA, smoke, alcohol drinking	TyG index was positively associated with baPWV (95% CI: 0.11–0.14)
Wu, 2021, China [52]	Cross-sectional study, 1895 prehypertensive patients	AS evaluated by baPWV	Age, sex, BMI, smoke, drinking, physical activity, DM, dyslipidaemia, FBG, TG, PBG, LDL-C, eGFR, UA, homocysteine, medications	TyG index was positively associated with baPWV (95% CI: 58.7–200.0)
Wang, 2021, China [53]	Observational study, 3185 patients with T2DM	AS evaluated by baPWV	BMI, waist circumference, blood pressure, LDL-C, HDL-C, WBC counts, smoke, drinking, medication	TyG index was positively associated with baPWV (OR: 1.40, 95% CI: 1.16–1.70)
Guo, 2021, China [54]	Observational study 13,706 healthy subjects	AS evaluated by baPWV, 10-year CVD risk was evaluated using the Framingham risk score	age, smoke, BMI, pulse pressure, HbA1c, TC, LDL-C, HDL-C, UA, medication	TyG index was independently associated with AS (OR: 1.514, 95% CI: 1.371–1.671) and 10-year CVD risk (OR: 1.420, 95% CI: 1.147–1.756)
Yan, 2022, China [95]	Cross-sectional study 2480 individuals from Hanzhong Adolescent Hypertension Cohort study	AS evaluated by baPWV	Age, sex, smoke, alcohol drinking, regular exercise, BMI, blood pressure, hs-CRP, diabetes	Higher long-term trajectory of TyG index were independently associated with increased arterial stiffness (OR: 2.760, 95% CI: 1.40–7.54)
Wu, 2021, China [96]	Prospective study 6028 individuals from Kailuan study	AS evaluated by baPWV	Age, se, smoke, alcohol drinking, physical activity, MAP, diabetes, hs-CRP, and BMI at baseline	TyG index was independently associated with AS (HR: 1.58, 95% CI: 1.25–2.01)
Coronary artery calcification				
Kim, 2017, Korea [57]	Observational study 4319 healthy subjects	CAC evaluated by multidetector CT scanner	Age, sex, blood pressure, BMI, LDL-C, HDL-C, smoke, alcohol, exercise habits	TyG index was independently associated with CAC (OR: 1.950, 95% CI: 1.23–3.11)
Won, 2020, Korea [58]	Observational study 12,326 asymptomatic subjects	CAC evaluated by multidetector CT scanner	Age, male sex, BMI, blood pressure, TC, TG, HDL-C, LDL-C, glucose, and creatinine, smoke	TyG index was significantly associated with CAC progression in baseline CACS ≤ 100
Park, 2019, Korea [97]	Retrospective study 1175 individuals	CAC evaluated by multidetector CT scanner	Age, sex, BMI, blood pressure, LDL-C, HDL-C, exercise, alcohol, smoking, presence of diabetes and hypertension, medication	TyG index is an independent predictor of CAC progression (OR: 1.82, 95% CI: 1.20–2.77)
Heart failure				
Guo, 2021, China [61]	Retrospective study, 546 patients with CHF and T2DM	Cardiovascular death or rehospitalization due to HF during follow up	Age, sex, BMI, blood pressure, HR, CRP, eGFR, NT-proBNP, HbA1c, LVEF, AF, NYHA	TyG index was positively associated with cardiovascular death (HR: 4.42, 95% CI: 1.49–13.15) and rehospitalization (HR: 1.84, 95% CI: 1.16–2.91)
Yang, 2021, China [63]	Retrospective study, 103 hospitalized HF patients	ECV fraction calculated by CMR measurements and T1 mapping, all-cause death or HF rehospitalization during follow up	age, DM, HbA1c, NT-proBNP	TyG index was the significant factor determined for ECV fraction (r partial = 0.36) and primary outcome events (HR = 2.01, 95% CI = 1.03–4.01)

*CAD* coronary artery disease, *T2DM* type II diabetes mellitus, *MACCE* major adverse cardiac and cerebrovascular events, *HDL-C* high density lipoprotein cholesterol, *UA* uric acid, *HR* hazard ratio, *CI* confidence interval, *CVE* cardiovascular events, *BMI* body mass index, *hsCRP* hypersensitive C-reactive protein, *LVEF* left ventricular ejection fraction, *OR* odds ratio, *LDL*-*C* low density lipoprotein cholesterol, *HbA1c* hemoglobin A1c, *CAS* coronary artery stenosis, *CACS* Coronary artery calcification score, *CT* computed tomographic, *Cr* creatinine, *CS* coronary stenoses, *CVRF* cardiovascular risk factor, *ACS* acute coronary syndrome, *MACE* major adverse cardiovascular events, *MI* myocardial infarction, *STEMI* ST-elevated myocardial infarction, *OCT* optical coherence tomography, *PR* plaque rupture, *PE* plaque erosion, *CABG* coronary artery bypass graft, *OPCABG* off-pump coronary artery bypass graft, *CKD* chronic kidney disease, *IABP* intra-aortic balloon pump, *eGFR* estimated glomerular filtration rate, *NSTE* non-ST-segment elevation, *GRACE* Global Registry of Acute Coronary Events, *MINOCA* MI patients with nonobstructive coronary arteries, *Mets* metabolic syndrome, *DES* drug-eluting stent, *ISR* in-stent restenosis, *SYNTAX* Synergy Between Percutaneous Coronary Intervention With Taxus and Cardiac Surgery, *LAD* left anterior descending artery, *RCA* right coronary artery, *CVD* cardiovascular disease, *IMT* intima-mediated thickness, *baPWV* brachial ankle pulse wave velocity, *AS* arterial stiffness, *FBG* fasting blood glucose, *PBG* postprandial blood glucose, *TG* triglyceride, *TC* total cholesterol, *CAC* coronary artery calcification, *TC* total cholesterol, *CHF* chronic heart failure, *HR* heart rate, *NT_proBNP* N-terminal B-type natriuretic peptide, *AF* atrial fibrillation, *NYHA* New York Heart Association functional classification, *PCI* percutaneous coronary intervention, *NOAF* new-onset atrial fibrillation, *Hb* hemoglobin, *WBC* white blood cell, *PVD* Peripheral vascular disease, *MAP* mean arterial blood pressure

### Stable coronary artery disease

Coronary artery disease (CAD) is one of the main causes of cardiovascular-related death. Although advanced therapeutics, including optimal drug strategies and revascularization, have effectively decreased the incidence of chest pain, patients with CAD still have an increased risk of experiencing major adverse cardiovascular events (MACEs) [21]. Consistent clinical data have suggested that an elevated TyG index is positively associated with poor outcomes in patients with CAD. A nested case–control study enrolled 1282 T2DM patients with new-onset stable CAD and revealed that an increased TyG index was associated with an increased risk of major adverse cardiovascular and cerebral events (MACCEs) after adjusting for confounding risk factors (HR: 1.693, 95% CI 1.238–2.316). Moreover, the addition of the TyG index to a Cox model containing glycated haemoglobin (HbA1c) was found to increase the predictive value for MACCEs [22]. A study by Jin et al. further confirmed the prognostic value of the TyG index in patients with stable CAD [23]. In addition, a single-centre observational study conducted by Gao et al. with a relatively large number of patients revealed the value of determining the TyG index (ORs: 1.59 and 5.72 in the T2 and T3 groups compared with the first tertile group) in patients with totally occluded coronary vessels over 3 months, namely, CTO lesions. Particularly, the improvement in the AUC value for the evaluation of less developed collateralization was most significant after adding the TyG index to the baseline model [24], providing novel information regarding the relation of TyG to clinical outcomes in patients with CAD (see Table 1).

In addition to the association with prognosis in patients with established CAD, the TyG index has also been used to identify asymptomatic patients with a high risk of atherosclerosis. Lee et al. enrolled a total of 888 asymptomatic adults with T2DM but without previous CAD to evaluate coronary artery stenosis (CAS) by coronary computed tomographic (CT) angiography and found that a higher TyG index was associated with an increased risk of CAS, similar to old age, male sex, poor glycaemic control, a longer duration of diabetes, and no statin use. Moreover, a higher TyG index was identified as an independent risk factor for CAD (OR: 3.19, 95% CI 1.371–7.424) [25]. A study by Thai et al. confirmed the role of the TyG index in identifying diabetic subjects at high risk of CAD in Vietnam. They found that the number of narrowed coronary arteries and the degree of coronary stenosis were also associated with a higher TyG index [26]. Nevertheless, the current guidelines for the primary prevention of CVD indicate that asymptomatic individuals without cardiovascular risk factors (CVRFs) are not considered candidates for preventive treatments [27]. Recently, a study by the Progression of Early Subclinical Atherosclerosis (PESA) indicated that in middle-aged populations without CVRF, the prevalence of subclinical atherosclerosis is approximately 50% [28]; therefore, identifying patients in this population who are at early risk for subclinical atherosclerosis is of great importance. In a retrospective observational study, Park et al. included 1250 asymptomatic Korean individuals without traditional CVRFs to evaluate coronary stenosis by coronary CT angiography. They found that the TyG index was associated with an increased risk of CAD (OR: 1.473, 95% CI 1.026–2.166), especially in patients with noncalcified and mixed plaques [29]. These studies support the notion that the TyG index is an independent marker that can be used to predict subclinical CAD both in general populations and individuals with established risk factors.

### Acute coronary syndrome (ACS)

ACS is the most severe type of ischaemic heart disease and describes a range of myocardial ischaemic conditions, including unstable angina (UA), non-ST elevated myocardial infarction (NSTEMI), and ST-elevated myocardial infarction (STEMI) [30]. Despite the use of current guideline-recommended therapeutics, including coronary artery revascularization techniques such as percutaneous coronary intervention (PCI) or coronary artery bypass grafting (CABG) and optimal drug treatments, some patients with ACS remain at high risk for recurrent cardiovascular events (CVEs) [31]. Thus, it is critical to identify ACS patients who are at a high risk of CVEs so that intense strategies can be provided. Studies have suggested that the TyG index might be a useful marker for risk stratification and for predicting the prognosis of ACS patients with or without diabetes. A retrospective cohort study enrolled a total of 2531 consecutive patients with established diabetes. These patients received coronary angiography (CAG) due to ACS and completed 3 years of clinical follow-up. The authors found that the incidence of MACEs increased along with the TyG index tertiles and that the TyG index was an independent predictor of MACEs (HR: 1.455, 95% CI 1.208–1.753) after adjusting for traditional CVRFs, irrespective of whether non-invasive or invasive treatments were administered [32]. However, in this study, subgroup analysis showed that the prognostic value of the TyG index was only significant in patients with UAP (adjusted HR: 1.604, 95% CI 1.270–2.027). One explanation for this result may be the small sample size. Subsequently, Luo et al. included 1092 STEMI patients who underwent PCI and found that the TyG index was positively associated with an increased risk of MACCEs in STEMI patients within 1 year following PCI after adjusting for confounding factors (HR: 1.529, 95% CI 1.001–2.061) [33]. Additionally, Mao et al. evaluated 438 patients with NSTE-ACS and followed them for 12 months after admission to assess the risk of MACEs. The results indicated that the TyG index presented strong diagnostic power for CVRFs, including glucose metabolism disorder and metabolic syndrome [34]. Furthermore, the TyG index was found to be an independent predictor of a high SYNTAX score (OR: 6.055, 95% CI 2.915–12.579) and the occurrence of MACEs (HR: 1.878, 95% CI 1.130–3.121). These two studies supported the potential value of using the TyG index for predicting clinical outcomes in patients with different groups of ACS. However, these previous studies were carried out only with patients who had an established DM diagnosis or impaired glucose tolerance. Is the TyG index also useful for predicting the prognosis of patients without glucose metabolic disorders? As discussed above, the TyG index has been reported to be useful for the early identification of apparently healthy individuals at high risk of developing CVD. Therefore, whether the TyG index can predict the clinical outcome of ACS patients without established risk factors may be of clinical interest. In an analysis of 1655 ACS patients without diabetes and low-density lipoprotein cholesterol (LDL-C) levels less than 1.8 mmol/L, Zhang et al. found that a high TyG index level was associated with a higher incidence of AMI (21.2% *vs.* 15.2%), larger infarct size and higher incidence of revascularization (8.9% *vs.* 5.0%) compared with ACS patients with LDL-C levels below 1.8 mmol/L. Interestingly, patients with a high TyG index were prone to develop DM during follow-up, indicating that they might be more likely to develop multivessel CAD, which would be a potential contributor to the increased incidence of revascularization [35]. The results of this study suggested that a high TyG index level might be a valid predictor for early stratification in ACS patients with relatively low risk.

In addition to obstructive ACS, an elevated TyG index is also independently associated with a poor prognosis in MI patients with nonobstructive coronary arteries (MINOCA). MINOCA is a distinct clinical entity and presents a heterogenous diagnosis of multiple causes, including plaque rupture or erosion, coronary spasm, thromboembolism, spontaneous dissection, microvascular dysfunction and supply/demand mismatch, accounting for 5–10% of all MI cases. Gao et al. recruited a total of 1179 MINOCA patients who completed a median follow-up of 41.7 months and found that the patients in the higher TyG index tertiles had an increased risk of MACEs (HR: 1.33, 95% CI 1.04–1.69) after adjusting for multivariate risk factors. Of note, the TyG index remained a robust risk factor in overall MINOCA patients or subgroups, including DM or non-DM patients and those with LDL-C levels higher or lower than 1.8 mmol/l, suggesting that the TyG was a reliable marker for predicting outcomes independent of glucose-lipid metabolic status in patients with MINOCA [36].

### In-stent restenosis

PCI is currently the most common revascularization strategy in Chinese patients with CAD, even those with diabetes. However, despite considerable improvements in outcomes due to the widespread use of drug-eluting stents, in-stent restenosis (ISR) remains one of the major challenges after PCI, occurring in 3–20% of patients [37, 38]. Therefore, the early identification of patients with a high risk of ISR may have great clinical importance. Zhu et al. retrospectively recruited 1574 patients who were admitted for ACS and underwent successful drug-eluting stent (DES)-based PCI. They found that an elevated TyG index was independently and positively associated with the occurrence of DES-ISR [39]. However, the incremental predictive value of the TyG index for DES-ISR was slight; thus, multicentre, large-scale clinical studies are necessary to clarify the relationship between the TyG index and ISR.

### Arterial stiffness

Atrial stiffness (AS) is one of the earliest types of functional damage that occurs during the vascular ageing process, during which the arterial elasticity decreases and pulse pressure increases [40, 41]. Mounting evidence has suggested that AS is a powerful predictor for the future risk of CVDs such as ACS, heart failure (HF), and ischaemic or haemorrhagic stroke [42, 43]. Considering that patients with AS suffer from long-term pathological progression, there is an urgent need for reliable biomarkers to identify patients in the early stage and to develop preventive therapeutics. In an analysis of 473 postmenopausal women without diabetes, Lambrinoudaki et al. showed a positive association between the TyG index and AS by measuring brachial ankle pulse wave velocity (baPWV). Nonetheless, this study was limited by its small size, and it included only postmenopausal women [44]. Subsequently, a Korean study enrolling 3587 healthy adults found that compared to the HOMA-IR index, the TyG index was independently associated with increased baPWV [45]. Won et al. [46] and Su et al. [47] provided further evidence to support the predictive value of the TyG index in identifying AS among healthy Korean adults and Chinese community-dwelling elderly individuals. Furthermore, mounting evidence has revealed that elevated baPWV is associated with an increased risk of hypertension [48] and diabetes [49], which are major risk factors for AS. Thus, it is of great importance to focus on the link between the TyG index and AS in different populations. Li et al. performed a study involving a large number of hypertensive adults and revealed that there was a significant positive association between the TyG index and baPWV (OR: 1.02, 95% CI 0.83–1.20), especially in men [50]. In contrast, Nakagomi et al*.* found that the association between the TyG index and increased levels of baPWV was stronger in women [51]. This discrepancy might be due to differences in the age distribution between these two studies; the mean age of individuals in Nakagomi et al. was 38.8 years old, while it was 64.41 in the study by Li et al. Therefore, further studies are needed to examine the relationship among IR, AS, sex and age. Recently, Wu et al. added more data to support the association of the TyG index with the progression of AS in hypertensive individuals. In their study, 1895 prehypertensive and hypertensive patients were followed up for a median of 4.71 years, and their results indicated that there was a linear and positive association between the TyG index and three baPWV parameters (baPWV change, baPWV change rate and baPWV slope) in hypertensive populations rather than in prehypertensive populations [52]. These results suggest that the interaction between IR and hypertensive status may contribute to AS development and progression; therefore, more attention should be given to IR indexes in patients with hypertension. In addition to hypertension, patients with diabetes can also develop AS. In a study involving 3185 patients with T2DM, Wang et al. showed a positive and dose–response relationship between the TyG index and AS, assessed by baPWV after adjusting for confounding factors (OR: 1.40, 95% CI 1.16–1.70). Moreover, compared to the HOMA-IR index, the TyG index was better at predicting an increased incidence of AS in T2DM patients [53], providing evidence to support that the TyG index can serve as a simple but reliable biomarker to evaluate AS in diabetic patients. Additionally, Guo et al. further demonstrated a positive association between the TyG index and 10-year CVD risk among a large number of patients with AS in China [54]. The results of all these studies reflect the potential value of the TyG index in predicting AS and in providing guidance to clinicians regarding appropriate treatment strategies.

### Coronary artery calcification

Coronary artery calcification (CAC), defined as an Agatston score > 0 by a multidetector CT scanner, is a sensitive marker for detecting the existence of early atherosclerosis. Additionally, CAC plays an important role in predicting adverse CVEs [55, 56]. Therefore, the identification of patients who have a high risk of CAC may have significant clinical relevance. A Korean study performed in 2016 was the first to explore the relationship between the TyG index and CAC in 4319 apparently healthy adults. The data showed that the TyG index was independently associated with the presence of CAC after adjusting for multiple risk factors (OR: 1.95, 95% CI: 1.23–3.11) [57]. In addition, Won et al. [58] enrolled a large number of asymptomatic healthy adults without severe CAC at baseline and demonstrated that a high TyG index was significantly associated with CAC progression, which was defined as a difference ≥ 2.5 between the square roots of the baseline and follow-up CAC scores (Δ√ transformed CACS) [58]. Notably, these two studies on the relationship between the TyG index and CAC were based on Korean healthy populations, which does not represent the characteristics of all patients with CAC. Thus, the significance of the TyG index for predicting CAC progression in individuals who have CAD still needs to be clarified.

### Heart failure

Epidemiological studies have demonstrated that heart failure (HF) is a growing health burden, with a prevalence of up to 1–2% in adult populations [59]. Recent studies have indicated that IR was the main cause for the poor prognosis of patients with HF [60]. Thus, the identification of IR surrogate markers would play a vital role in the prevention and treatment of HF. Guo et al. showed that the TyG index was positively related to the prognosis of patients with chronic HF and DM. They revealed that the higher the TyG index is, the higher the risk of cardiovascular death or rehospitalization caused by HF [61]. In addition to predicting the prognosis of patients with HF, the TyG index was also identified as a novel biomarker of cardiac fibrosis in these patients. The myocardial fibrosis estimated by cardiovascular magnetic resonance (CMR) can provide important prognostic information on the cardiovascular risk of HF [62]. Yang et al. analysed 103 hospitalized HF patients and found that myocardial fibrosis could quantified by the extracellular volume (ECV) fraction using CMR. Multivariate regression linear analysis showed that the TyG index was a significant determinator for the ECV fraction (*r*_partial_ = 0.36) in patients with HF. Additionally, during a median follow-up of 12.3 months, the TyG index was identified as an independent risk factor for all-cause mortality and HF hospitalization (HR: 2.01, 95% CI 1.03–4.01), supporting the utility of the TyG index in stratification metrics during the management of HF [63].

## Potential explanations of the TyG index as a marker for predicting cardiovascular disease

**Fig. 3:**
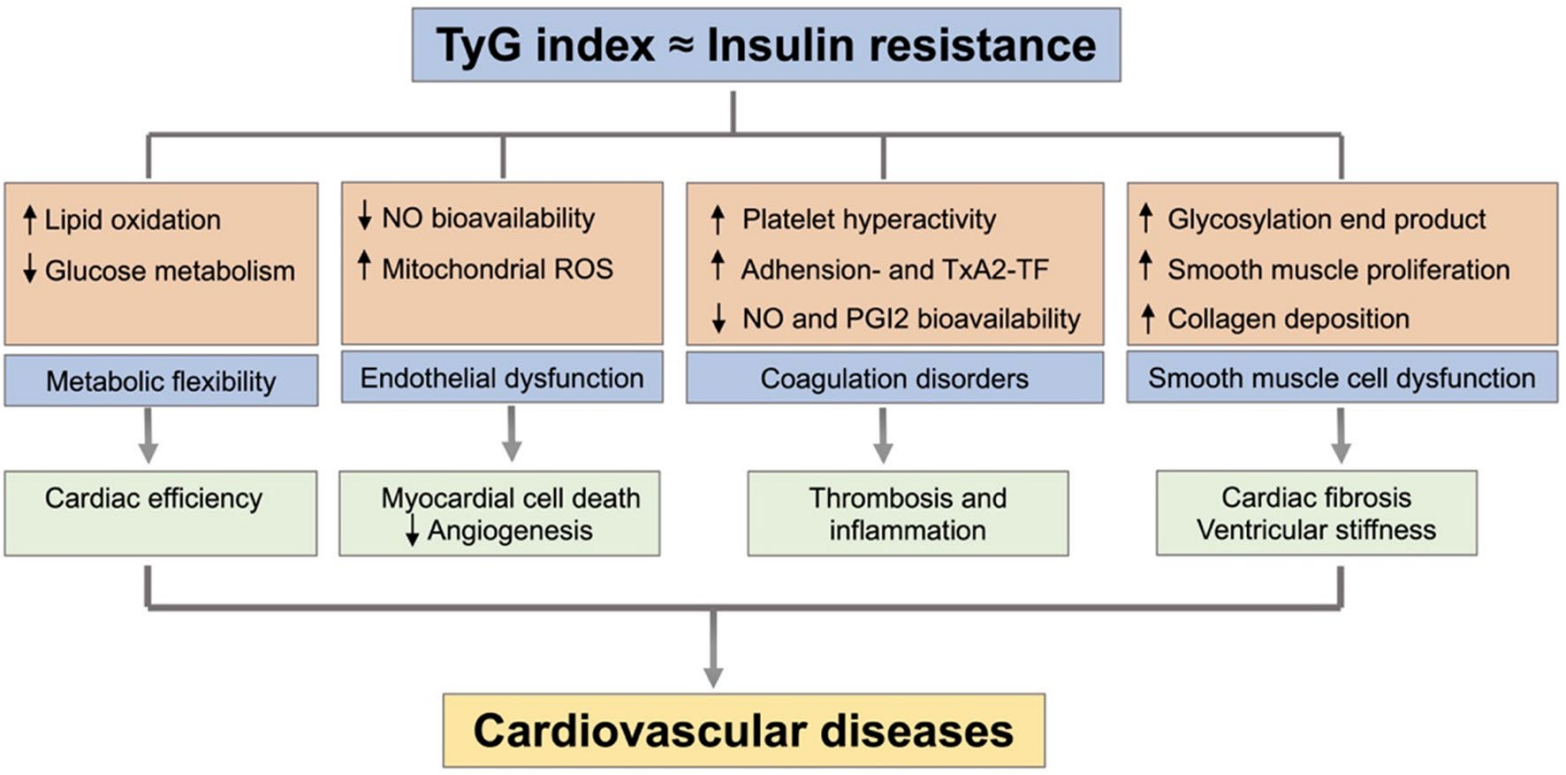
Potential molecular mechanisms that contribute to the predictive role of the triglyceride-glucose (TyG) index in cardiovascular diseases (CVD). Insulin resistance (IR) is a hallmark of metabolic syndrome and has been evidenced as a risk factor for CVD. The TyG index has been identified as a reliable alternative marker of IR, which may explain the association between the TyG index and CVD. The molecular mechanisms underlying IR and CVD include metabolic flexibility, endothelial dysfunction, coagulation disorders and smooth muscle cell dysfunction. TyG: triglyceride-glucose; CVD: cardiovascular diseases; IR: insulin resistance; NO: nitric oxide; ROS: reactive oxidative stress; TxA2: thromboxane A2; TF: tissue factor; PGI2: prostaglandin I2

The exact mechanism underlying the relationship between the TyG index and CVD remains unknown. It is very clear that TyG is an index consisting of two risk factors for CVD, lipid-related and glucose-related factors, which are reflective of IR in the human body. Recent studies have identified the TyG index as a reliable marker of IR, which may be one of the explanations for this association [15]. IR is a risk factor for CVD, which not only leads to the development of CVD in both the general population and patients with diabetes but also predicts the cardiovascular prognosis of patients with CVD [7]. The potential mechanisms underlying IR and CVD are described as follows (Fig. 3).

First, IR can induce glucose metabolism imbalance, contributing to hyperglycaemia, which in turn triggers inflammation and oxidative stress. Additionally, systemic lipid disturbances have also been reported, including elevated TG, small dense LDL, and postprandial lipaemia levels and reduced high-density lipoprotein (HDL) levels, which may cause the initiation of atherosclerosis [64]. Moreover, in established ischaemic myocardium, reduced insulin activity limits glucose bioavailability and causes a shift to fatty acid metabolism, ultimately leading to increased myocardial oxygen consumption and a reduction in the compensatory capacity of non-infarcted myocardium [65]. These pathological metabolic disorders further aggravate CAD progression.

Second, studies have shown that IR can induce an increased production of glycosylated products and free radicals, leading to nitric oxide (NO) inactivation. The abnormal secretion of NO related to IR damages the vascular endothelium and causes endothelium-dependent vasodilation [66]. Furthermore, IR also activates the mitochondrial electron-transport chain and induces overproduction of reactive oxidative stress (ROS), which is another cause of impaired endothelial function [67]. The abnormal endothelial function observed in patients with diabetes extends to the coronary microcirculation and myocardial energy metabolism. In patients with cardiac ischaemia, IR is inversely associated with median colony forming unit endothelial cells, contributing to a reduced density of collaterals in response to cardiac ischaemia [68].

Moreover, many experimental studies have clearly established that the insulin receptor can mediate related signalling to sensitize platelets to the antiaggregating actions of prostaglandin I2 (PGI2) and NO. On the one hand, IR may contribute to platelet hyperactivity. On the other hand, it can increase adhesion-induced and thromboxane A2 (TxA2)-dependent tissue factor expression in platelets. These events have been implicated in both thrombosis and inflammation [69], which may partly explain the obstructive ACS or nonobstructive coronary thromboembolism observed in some patients.

In addition, previous studies have demonstrated that IR, which is usually accompanied by hyperglycaemia, induces excessive glycosylation, which can promote smooth muscle cell proliferation, collagen crosslinking, and collagen deposition. These pathological events then contribute to increased diastolic left ventricular stiffness, cardiac fibrosis and, ultimately, heart failure [7].

Finally, in addition to its role in hyperglycaemia, IR plays an important role in hyperlipidaemia. Studies have suggested that increased TG levels can induce elevated free fatty acid (FFA) levels and promote the increased flux of FFAs from adipose tissue to non-adipose tissue, which may accompany IR [70]. More importantly, the retention of cholesterol-rich and TG-rich ApoB-containing remnants within the coronary wall may be considered related to the pathogenesis of atherosclerosis [71]. Thus, lowering TG levels appears to be an additional target in patients with a high CVD risk. Additionally, activation of the renin-angiotensin system [72] and impaired cardiac calcium processing capacity [73] may also be contributors.

## Limitations of the TyG index as a marker in cardiovascular diseases

The TyG index is a composite indicator composed of fasting TG and FG, which could be used as an alternative test for recognizing IR in large-scale studies or for evaluating populations at high risk of developing diabetes. Notably, several studies have suggested that the TyG index was better than the HOMA-IR index in predicting the development of atherosclerosis and poor outcomes such as the increased occurrence of carotid atherosclerosis [74] and CAC progression as evaluated by the CAC score [75]. Moreover, according to previous studies, the direct qualification of serum insulin levels is expensive and is not available in most cities in developing counties; an alternative test derived from fasting TG and FBG is less costly and universally available. In addition, due to the need for quantitation, exogenous insulin may interfere with the value of the HOMA-IR index. Therefore, the current evaluation of IR by the HOMA-IR index may not be applicable to diabetic patients who are treated with insulin. Since the TyG index is a formula composed of fasting TG and FG, it does require the quantification of insulin and thus may be widely applicable in all diabetic patients treated with insulin. In summary, TyG is regarded as an accessible and reliable index for IR in individuals with a high risk of CVD, especially in developing counties.

However, there are still several observations that have failed to support the association between the TyG index and CVEs. First, the rationale for the first use of the TyG index in 2008 was that IR is a common cause of the increase in TG and glucose levels in healthy individuals [9]. Therefore, the application of the TyG index in CVD patients can be affected by hyperlipidaemia and diabetes. To justify the value of the TyG index as a biomarker, hypertriglyceridaemia and glucose metabolic disorder should be well controlled. Nevertheless, several patients with extremely high TGs or FBSs were still enrolled in previous clinical studies, which could not explore reverse causality in the application of the TyG index in these CVD patients. For example, Laura et al. did not find an association between the TyG index and CVD in subjects with T2DM or hypertension at baseline. Their outcomes could be explained by the hypothesis that patients previously diagnosed with diabetes or hypertension were under treatment or had adopted healthier habits, so their analytical parameters might be well controlled [20]. Cho et al. also failed to find an independent association between the TyG index and the presence of CAD or obstructive CAD in 996 patients with established diabetes after adjusting for traditional CVRFs [76]. Unfortunately, in this study, detailed information regarding the doses used, classes of patients enrolled and eventual changes in related drugs was unavailable. Hence, the potential influence of medications taken for hyperlipidaemia, diabetes and hypertension could not be excluded in these studies. Finally, other important information, including physical activity, alcohol consumption, and family history of diseases, was also lacking in many clinical studies.

Second, medical doctors involved in clinical work usually first pay attention to FBG and TG levels when screening patients with a high risk of CVD. However, the question of how the TyG index can add to the predictive value of TG and FBG levels remains. The comparison of the predictive values between the TyG index and TG and FBG (and may be the combination) is also missing in some studies. In addition, CVD is a series of dynamic and progressive disturbances, and the development of acute diseases such as MI may lead to stress hyperglycaemia, which may affect the diagnostic or predictive value of the TyG index based on the TyG formula. In most studies, TG and FBG were examined only at baseline, regardless of their changes over time, which may lead to potential regression dilution bias. Therefore, the measurement of the TyG index at baseline alone does not reflect the longitudinal association between the TyG index and CVD risk over time. Recently, Cui et al. showed that the risk of CVD development increased along with the quartile of the cumulative TyG index (defined as the summation of average TyG index for each pair of consecutive evaluations multiplied by the time between these two consecutive visits in years), showing a multivariate-adjusted HR of 1.39 (95% CI 1.21–1.61) [77]. These authors found that the cumulative effect of the TyG index seemed to be independent and better than the TyG index at baseline in predicting CVD. Therefore, the use of the TyG index at baseline as a biomarker to predict outcomes of CVD may be less robust. Evaluating the mean changes in the TyG index in CVD progression and follow-up is warranted in future studies.

Moreover, most studies regarding the use of TyG in CVD have been performed in middle-aged or elderly individuals, and no data are currently available concerning the value of TyG in young subjects. Dikaiakou et al. found that the TyG index showed a positive correlation with IR among both children and adolescents [78]; however, data regarding the predictive ability of the TyG index in identifying the presence of future CVD in these younger individuals are limited. In addition to the lack of information on different age groups, differences in the TyG index between the sexes are also still uncertain. Compared to women, men have more risk factors for metabolic diseases. For example, men are more likely to smoke and drink and have higher serum uric acid and serum homocysteine levels and a lower estimated glomerular filtration rate (eGFR) [51]. Therefore, further sex-related studies are warranted to explore the relationship between the TyG index and CVD. Finally, dietary habits can dramatically affect TG levels. However, nutrition data are missing from most studies, so we were unable to adjust for dietary habits when evaluating the diagnostic or predictive value of the TyG index in CVD.

## Conclusions

Overall, IR, a well-established hallmark of metabolic disorders and systemic inflammation, is not only a substantial risk factor for CVD but also contributes to a worse prognosis. Current studies have confirmed that the TyG index can be used as a reliable and convenient surrogate for IR, which may be optimized for risk stratification as well as outcome prediction for CVD. Nevertheless, based on current studies, there are some knowledge gaps that need to be addressed. First, some investigators have proposed that it would be interesting to explore whether a postprandial TyG index might have clinical significance. Because increased postprandial levels of TG and glucose are metabolically abnormal responses to IR, an elevated postprandial TyG index may be associated with a higher risk of diabetes or CVEs, which remains to be clarified. Second, regarding the predictive power of the TyG index in CVD, especially in CAD, accumulating studies have shown that the predictive value of the TyG index for CAD is mild to moderate, suggesting that it is difficult to predict severe CVEs based on the TyG index alone. Nevertheless, Wang et al. and Zhu et al. demonstrated that when introducing the TyG index into an established risk model, TyG could significantly improve the predictive accuracy for MACEs in patients with ACS [32, 39]. Thus, routinely adding the TyG index into clinical diagnostic models might help to refine cardiovascular risk stratification and enable the administration of more targeted therapeutics or prevention measures. Finally, the pathological role of the TyG index in different types of CVD still warrants further research. The potential benefits of TyG index-targeted treatments in CVD patients also require more in-depth validation.

## Data Availability

Not applicable.
